# Cognitive Functioning in Healthy Females with Different Levels of Cognitive Reserve

**DOI:** 10.1002/alz70860_103165

**Published:** 2025-12-23

**Authors:** Una Lazic, Predrag Aleksić, Maksim Šarčević, Isidora Dzodic, Anja Vrlješ, Ana Lešić, Biljana Salak‐Djokic, Vera Ilic, Gorana Mandic Stojmenovic, Elka Stefanova, Tanja Stojkovic

**Affiliations:** ^1^ Neurology Clinic, University Clinical Center of Serbia, Beograd, Serbia, Serbia; ^2^ Neurology Clinic, University Clinical Center of Serbia, Belgrade, Belgrade, Serbia; ^3^ Neurology Clinic, University Clinical Centre of Serbia, Belgrade, Serbia; ^4^ Faculty of Medicine, University of Belgrade, Belgrade, Serbia; ^5^ Neurology Clinic, Clinical Center of Serbia, Belgrade, Serbia

## Abstract

**Background:**

Women are disproportionately affected by Alzheimer's Disease (AD), with sex being the second strongest risk factor after age. Consequently, over 65% of people with late‐onset AD are women. Therefore, a preventative approach is especially important in the female population. Cognitive reserve (CR) is recognized as a strong protective factor against AD. Its importance for cognitive functioning is reflected by the fact not taking it into account can have a clinical implication of lack of good randomization and unwanted skewing of clinical trial results. The studies exploring relationships between CR and cognitive performance in healthy female population are scarce. This study aimed to explore the differences in cognitive functioning in working‐age women with different levels of CR.

**Method:**

A total of 114 healthy female volunteers aged 30‐65 years were recruited among employees of the University of Belgrade. The exclusion criteria included conditions affecting communicative ability and/or safe engagement in the interventions, as well as the presence of any neurological, psychiatric, medical condition or iatrogenic cause known to affect the brain structure and/or function. All participants underwent a detailed assessment including basic demographic data, vascular risk factors, mood scales and comprehensive neuropsychological assessment as well as Cognitive Reserve Index Questionnaire (CRIq). Statistical analysis was performed using SPSS20.

**Result:**

The mean age of our subjects was 48.11±0.80 years with mean education of 19.01±0.39 years. CRI levels were classified as medium (28.1%), medium‐high (34.2%), and high (37.7%). Regarding neuropsychological testing, significant differences were found on tests of category fluency (*p* = 0.002) and visuospatial domain including visual memory (Rey–Osterrieth complex figure copy (*p* = 0.002), spatial recognition memory percent correct (*p* = 0.023), rapid visual processing (*p* = 0.026)), with the group with the lowest CRI having the poorest results. These differences withhold correction for age.

**Conclusion:**

CR interacts with cognitive abilities even at working age in women. This interaction is most significant in domains that are predominantly affected in AD. These results support the theory of strong protective effect of CR in this at‐risk population. Opportunities for CR development are especially important in female gender and should be tailored into preventative AD strategies.

